# Journal, Association, and Public Health in Medical Colleges

**DOI:** 10.4103/0970-0218.62543

**Published:** 2010-01

**Authors:** Rajesh Kumar

**Affiliations:** PGIMER School of Public Health, Chandigarh 160 012, India E-mail: dr.rajeshkumar@gmail.com

Journals published by Professional Associations play an important role in scientific literature. However, the relationship between the Journal and the Association is quite complex. Notwithstanding the legal and economic realities, the Associations should respect independence of the Journal. The Committee on Publication Ethics (COPE) has rightly mentioned in their guidelines that the relationship of the editors of the journal and the Association should be firmly based on the principle of editorial independence. In case the Association, Authors or Reviewers are not satisfied with the decision of the editors, the Journal should have mechanisms such as an Ombudsman to resolve issues, without unnecessarily divulging the sensitive information. However, Associations generally do not encourage creation of these mechanisms, lest they lose ‘control’ over their Journal.

The future augurs well for the Indian Journal of Community Medicine. Submissions to the Journal have increased to nearly five-fold, revenue has gone up four times, it now receives articles online and publishes many more pages on high quality paper, in multicolor. Moreover, it is now indexed in the PubMed. These developments will motivate authors to send their best research work to the Indian Journal of Community Medicine. A solid base of subscribers built over the years will sustain it financially. A strong peer review process has been instituted, to select the best manuscripts for publication. Using the key words of the submitted manuscript, a search is made in the PubMed for selecting at least six reviewers from among the authors who have published on the topic in the recent past, so that editors get comments from at least two of them. In general, the Journal has done well; however, there are many pressures that can deflect it from its course. The health of the Journal is the reflection of the health of the Association, and if the Journal ever collapses it will be because the Association did not care for it.

In the competitive era of scientific publications, it is important to keep the clients, that is, subscribers and authors happy by timely decisions and publication. Unfortunately, due to the delays in getting the submissions peer-reviewed, the decision-making process gets slowed down, causing a lot of frustration among the authors. The submission to decision time needs to be decreased to less than three months, and the accepted articles should be published within the next six months. These goals can be achieved by implementing the policy of in-house rapid review by the editorial team. In this manner, the editorial team can quickly decide whether to send the submission for external peer review or decline publication. However, the objectivity in the selection process should remain supreme, and the criteria that guide the selection process must be to find out whether the manuscript reports newly created knowledge. The success of the Journal lies in its ability to attract articles that have the potential to improve the practice of community medicine.

The contents of the past issues of the Journal, recently archived on its website, are a reflection of how vibrant the specialty of preventive/social/community medicine has been, and of the role it has played in improving the health status of the Indian population. Of course the teaching of preventive/social/community medicine in medical colleges has helped in broadening the worldview of hundreds and thousands of physicians and surgeons, who currently occupy administrative positions, practice in the community, teach in medical colleges or conduct research. Several of them have taken up the cause of public health, despite specializing in other medical or surgical specialties. Rather than despising them, we must be proud of them for championing the cause of public health.

We should also advocate for enhancing the role of medical colleges in public health. Community Medicine Departments must evolve into a multidisciplinary faculty, covering all aspects of public health. In a short-term, related faculty members from other institutions/universities can be appointed as part-time/honorary/guest faculty to enrich the teaching/learning process. It will also help in broadening the research agenda, to better understand the health problems from multiple angles. The contents/curriculum also needs to be updated, to broaden the biomedical understanding of the graduates to a sociological approach that emphasizes issues related to equity, ethics, justice, and the need for advocating healthy public policies in every sector.

Placement of faculty and students in other organizations in the government and non-government sectors, which have a good track record, must be considered, so that they have opportunities of practicing public health in real-life situations. Medical colleges should have a good collaboration with the government and non-government organizations, at least in the area where they are located. These collaborations are mutually beneficial, but difficult to manage. Memorandum of Understanding (MoU) with clearly defined roles and responsibilities of each organization and establishment of a coordination mechanism with periodic review at a higher level can facilitate these initiatives. Community Medicine Specialists should spearhead the movement to place public health as a central focus of medical colleges with a mission to improve the health of the community rather than only teaching and treating those who enter into their portals.

With this issue I step down from the chief editorship of the Journal. I express my appreciation to our advisers and the editorial board members, particularly to my colleagues in the editorial team, especially the Managing Editor, who have brought it all together, working voluntarily for the last six years, and upholding the science ahead of personal ambitions. I also thank the authors and reviewers who are the prime architects of the quality of its contents. It has been a professionally rewarding and exciting experience for me, which has inevitably earned me a few enemies! although, many more friends. I wish the new editorial team well and hope that they will take the Journal to new heights.

